# A National Survey on Perineal Reconstruction Following Standard and Extralevator Abdominoperineal Excision: Current Practices and Trends in the UK

**DOI:** 10.7759/cureus.28339

**Published:** 2022-08-24

**Authors:** Rushabh Shah, Rituja Kamble, Mohammed Herieka, Milind Dalal

**Affiliations:** 1 Plastic and Reconstructive Surgery, Manchester University NHS Foundation Trust, Manchester, GBR; 2 Plastic and Reconstructive Surgery, University Hospitals Plymouth NHS Foundation Trust, Plymouth, GBR; 3 Plastic and Reconstructive Surgery, Lancashire Teaching Hospitals NHS Foundation Trust, Preston, GBR

**Keywords:** uk, survey, extralevator abdominoperineal excision, abdominoperineal resection, perineal reconstruction

## Abstract

Background

Challenging perineal defects resulting from extralevator (ELAPE) and standard abdominoperineal excision (APE) have given rise to an emerging multidisciplinary approach between colorectal and plastic surgeons. At present, there is a relative paucity of evidence on best practice. This study sought to assess current national practice concerning perineal reconstruction following APE/ELAPE in the United Kingdom (UK) and to determine the factors involved in reconstruction choice.

Methodology

An anonymised survey was circulated to consultant plastic surgeons at all 48 UK centres performing perineal reconstruction following APE/ELAPE. Responses were collected between October 2021 and April 2022.

Results

Complete responses were received from 24 units nationally. All units had a dedicated APE/ELAPE service. Overall, 70% adopted a standardised reconstructive approach, the most common being the inferior gluteal artery perforator flap (n = 11). Significant variation was identified in the reconstructive technique. Similar differences were observed in the perceived importance of surgical factors guiding the reconstructive decision-making process, the top priorities being the size of the defect and previous radiotherapy.

Conclusions

The variability of responses suggests a lack of national consensus on optimal reconstruction following APE/ELAPE, despite the majority of centres employing a standardised approach to reconstruction. Our study highlights important surgical decision-making factors and provides valuable insight to aid in developing national collaborative evidence-based guidelines on best practice.

## Introduction

Management of low rectal and anal carcinomas may involve abdominoperineal excision (APE); however, in recent years, its popularity has reduced due to reported poorer survival and higher local recurrence rate compared with anterior resection [1,2]. A modified technique, extralevator abdominoperineal excision (ELAPE), is increasing in popularity for the treatment of locally advanced rectal carcinomas due to its association with reduced risk of local recurrence and improved survival [2-4]. The latter involves extensive en-bloc excision of the anorectum with the levator ani muscles, which significantly reduces the risk of circumferential resection margin involvement and intraoperative tumour perforation [5,6].

Consequently, patients undergoing ELAPE are left with a larger perineal defect with a greater volume of dead space compared with standard APE [7,8]. Use of neoadjuvant chemoradiotherapy to improve survival further increases the risk of perineal wound complications [9-11].

Challenging perineal defects resulting from ELAPE and standard APE have resulted in a growing multidisciplinary approach between colorectal and plastic surgeons. At present, several approaches for perineal reconstruction have been described, including primary closure, biologic mesh reconstruction and flap-based procedures [12,13]. However, there is a paucity of evidence on best practice and the factors informing reconstructive planning after APE/ELAPE.

The aim of this study was to survey current practice in the United Kingdom (UK) and determine the factors involved in the reconstructive decision-making process.

## Materials and methods

An anonymous online survey was designed using Google Forms and circulated via email in October 2021 to all substantive consultant plastic surgeons across all 48 UK centres performing perineal reconstruction following APE/ELAPE. Direct telephone enquiries were made to all hospitals listed on the British Association of Plastic and Reconstructive Surgeons (BAPRAS) database to ascertain whether perineal reconstruction is performed at the unit, and contact details of the relevant consultant plastic surgeons were obtained. A single response was requested from each centre to represent the unit’s practices. Surgeons who did not respond to the initial email request were sent a maximum of two reminders over one-month intervals. Responses were collated between October 2021 and April 2022. No formal ethical approval was required for the survey.

The survey was designed to cover the following domains (see Appendix 1): general demographics; scope of practice; choice of perineal reconstruction; and factors involved in decision-making.

Microsoft Excel (Office 2019, Microsoft Corp., Redmond, WA) was used for data analysis. Data from all responses, including incomplete forms, were anonymised and included in the final analysis. Results are displayed as frequencies and/or percentages.

## Results

A total of 48 units across the UK perform perineal reconstruction following APE/ELAPE. Responses were received from 24 units (a response rate of 50%), of which 22 (91.7%) completed the survey in its entirety.

General demographics

**Figure 1:**
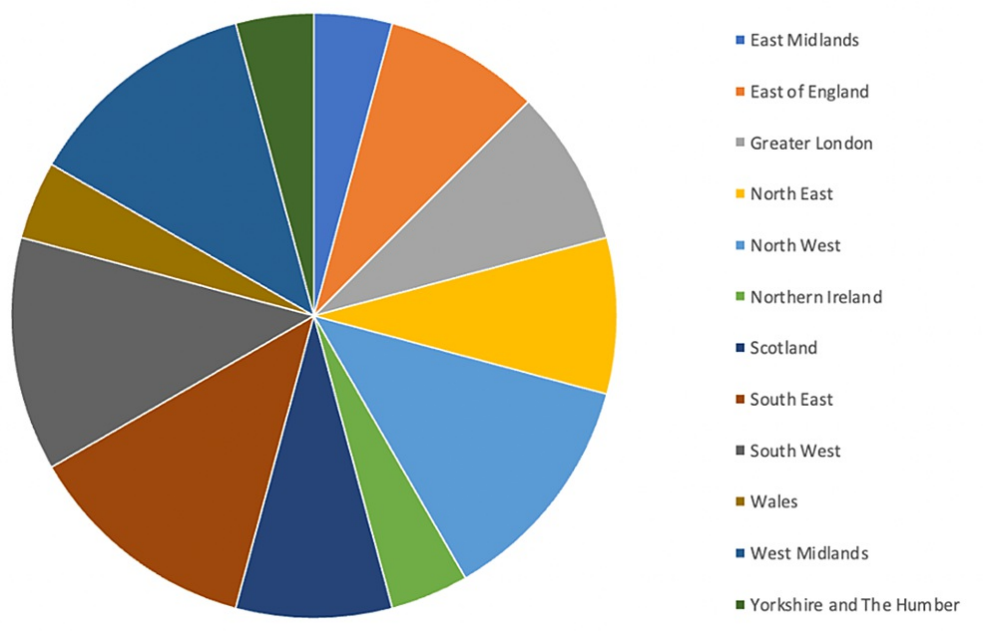
Percentage of responses by region.

Responses were received from units within all 12 regions in the UK (see Figure 1): Greater London; South-East; South-West; East of England; West Midlands; East Midlands; North-West; North-East; Yorkshire and The Humber; Scotland; Wales; and Northern Ireland. All respondents worked in plastic surgery. The vast majority of respondents were consultants (91.7%, n = 22), with the remaining being senior registrars (8.3%, n = 2). All respondents had a plastic surgery unit available at their hospital.

Scope of practice

**Figure 2:**
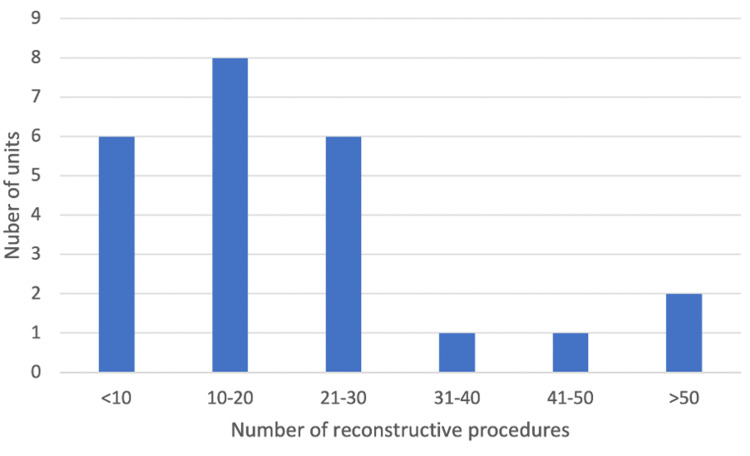
The number of perineal reconstruction procedures performed by each unit in the preceding year.

All units had a dedicated APE/ELAPE service. There was a wide range of reconstructive procedures being performed by each unit per year (see Figure 2), with the majority of units performing between 10 and 20 procedures. Most units adopted a standardised approach to reconstructing perineal defects following APE/ELAPE (70%, n = 16).

Choice of perineal reconstruction

**Figure 3:**
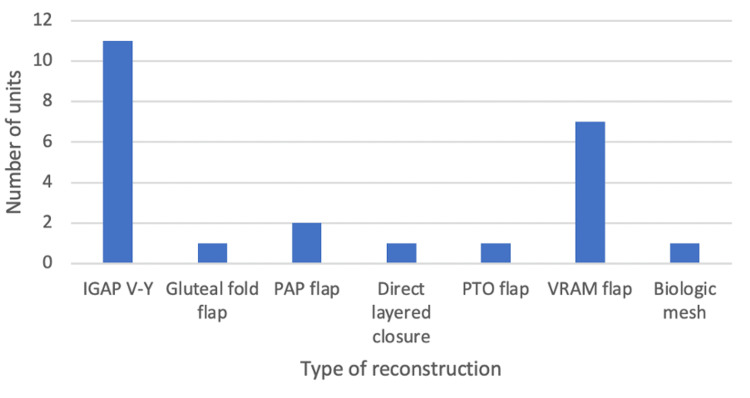
The preferred approach for reconstructing perineal defects following APE/ELAPE. APE/ELAPE: extralevator abdominoperineal excision/abdominoperineal excision

Overall, there were seven different preferred approaches to reconstruction including (see Figure 3) direct layered closure; perineal turnover (PTO) flap; inferior gluteal artery perforator (IGAP) V-Y advancement flap; interposition using biological mesh; vertical rectus abdominus musculocutaneous (VRAM) flap, profunda artery perforator (PAP) flap; and gluteal fold flap. The most commonly preferred approach was the IGAP V-Y flap (45.8%, n = 11), with the second most common being the VRAM flap (29.2%, n = 7).

Approximately two-thirds of the respondents (62.5%, n = 15) performed the reconstruction with the patient in the prone position. The majority of the respondents routinely turned their patients intraoperatively (70%, n = 16).

**Figure 4:**
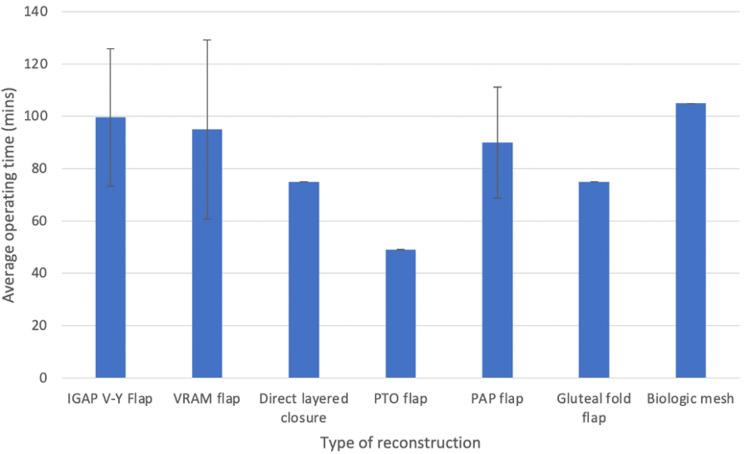
Average estimated operating time by reconstructive approach (minutes).

Significant differences were noted in the estimated operating time for the reconstruction of choice (see Figure 4), with the PTO flap being the quickest (45 minutes), and interposition with biological mesh being the slowest (120 minutes). The majority of units did not routinely use mesh/acellular dermal matrices (ADM) for pelvic floor reconstruction (95.8%, n = 23).

Factors involved in decision-making

Overall, six factors were noted to play a key role in decision-making: the size of the defect; previous radiotherapy; patient habitus; previous abdominal surgery; intraoperative positioning; and cosmetic outcome. Respondents reported defect size and previous radiotherapy to the operative field as the two most important factors influencing reconstructive choice. Intraoperative positioning and cosmetic outcomes were deemed to be the two least important factors.

## Discussion

Perineal defects following APE/ELAPE pose a reconstructive challenge for plastic surgeons, whose assistance in perineal wound closure is sought in 67% of cases [14]. Perineal wound complications (e.g., seroma, dehiscence, perineal hernia, pelvic abscess, fistulae, or sinus formation) can cause significant patient morbidity [15]. Among the majority of colorectal surgeons, ELAPE has become the surgical treatment of choice for low rectal and anal carcinomas [15-17]. Although ELAPE is associated with superior oncological outcomes compared with standard APE, it involves creating a larger pelvic cavity with significantly higher wound-related complication rates [1]. In addition, the use of neoadjuvant chemoradiotherapy for rectal carcinomas may worsen perineal wound morbidity rates [14].

Numerous techniques of perineal reconstruction following APE/ELAPE have been described. These include direct layered closure, the interposition of biological mesh, and myocutaneous and fasciocutaneous flaps; however, there is a lack of consensus on the optimal reconstructive method. Several factors play a role in deciding the reconstructive approach including patient factors (e.g., age, gender, comorbidities, smoking status) and surgical factors (e.g., patient habitus, previous radiotherapy, previous abdominal surgery, size of the perineal defect, cosmetic outcomes, intraoperative positioning). In our results, the four most significant surgical factors were the size of the defect, previous radiotherapy, patient habitus, and previous abdominal surgery, respectively. Cosmetic outcome was deemed to be the least important factor influencing the reconstructive decision-making process.

The vast majority of plastic surgeons in our survey (92%) preferred a flap reconstruction for perineal defects as their standard approach. Although the majority of perineal defects following APE/ELAPE are primarily closed by colorectal surgeons [14,16], several studies have demonstrated the superiority of flap-based reconstructions over direct layered closure, particularly in the context of previous radiotherapy [15,18,19]. There is a high wound infection and dehiscence rate of up to 50% [2], and perineal hernia rate of up to 26% [20], associated with direct layered closure after APE. This suggests that primary closure alone may be insufficient in addressing the perineal defect.

Only one plastic surgery unit in our study preferred using primary closure with biological mesh (such as Permacol®) as their standard reconstructive approach, which is significantly lower than the national APE registry where up to 55% of perineal defects following ELAPE were closed using mesh [14]. Biological mesh is not vascularised or autologous tissue. It is prone to infective wound complications of up to 20% and is associated with an increased incidence of perineal pain of up to 43% [15,21]. Additionally, reconstruction with biological mesh was associated with the longest operating time in our study, which is in contrast to current literature [8]. Although limited, current literature suggests no significant difference in perineal wound morbidity between biological mesh and myocutaneous flaps, with the added advantage of shorter operating times and reduced hospital stay [8]. However, in the absence of high-quality prospective trials, the validity of these results is yet to be determined.

Myocutaneous flaps, such as VRAM and gracilis flaps, have been traditionally considered workhorse flaps for perineal defects [22]. VRAM flap was the second most preferred (29%) method of reconstructing perineal defects following APE/ELAPE in our study, while the gracilis flap was not preferred by any of the units as a standard reconstructive option. Abdominal-based flaps, such as the VRAM, offer the advantages of tissue bulk, ease of dissection, reliable vascular pedicle, and provision of vascularised tissue from non-irradiated areas in patients who have had prior radiotherapy. Several studies have also demonstrated favourable perineal wound complication rates compared to primary closure and certain thigh-based flaps (PAP flap and gracilis flap) [12,19,23,24]. However, the need for a significant abdominal incision precludes the use of the VRAM flap in laparoscopic or robotic-assisted APE/ELAPE, both of which have become more favourable in recent years [14]. Additionally, with up to 90% of perineal resection in ELAPE being performed in the prone position [14], there is an additional requirement to turn the patient intraoperatively (60% of units preferring VRAM turned patients intraoperatively in our study). Other significant limitations of the VRAM flap include the risk of partial/total flap necrosis [12], significant donor site morbidity [25], and disruption of potential future stoma sites [24], the latter being an important consideration in younger patients who have a higher likelihood of developing parastomal hernia and need for stoma re-siting [26]. The use of the VRAM flap is limited in high body mass index patients, where flap raise and inset can be challenging due to the significant soft-tissue bulk.

The most preferred reconstructive option reported by respondents in our study (45.8%) was the IGAP V-Y flap which has gained popularity in recent years as an alternative to the VRAM flap. The IGAP flap has been shown to provide robust soft-tissue coverage, with minimal perineal wound complications [7,22]. Additionally, the IGAP can be performed with the patient prone or in any of the variations of the Lloyd-Davies position. It also avoids the significant donor morbidity associated with sacrificing the rectus abdominus compared with VRAM flaps. The main drawback of this flap is the relatively longer reported procedure time. Our study found that the IGAP had the longest operating time among all flap reconstructive options (an average of 100 minutes), which is corroborated by reports in the current literature [7,22].

Interestingly, our study found that the PTO flap was preferred only at a single centre but was associated with the lowest mean operating time (an average of 49 minutes). Additionally, the PTO flap is associated with minimal wound complication rates and very low donor site morbidity [27,28]. Similar to the IGAP, it can be performed in the prone or Lloyd-Davies position, thus reducing the need for intraoperative turning, and does not interfere with stoma formation [27]. Furthermore, the internal pudendal artery perforators (which form the basis of the flap) lie outside the irradiation zone, allowing this flap to be used safely in patients who have had previous radiotherapy [28]. The main disadvantage of this flap is the reduced tissue bulk, which limits its use in very large cutaneous defects.

A recent review by Copeland-Halperin et al. has suggested that perineal-based flaps (such as the IGAP and PTO flaps) are associated with reduced perineal wound complications and patient morbidity [24]. However, these results were based on small, non-comparative, retrospective case series. A lack of prospective comparative studies in the current literature between different flap options makes direct comparisons difficult.

The limitations of this study are inherent to all questionnaire-based surveys. To provide a snapshot of an individual unit’s practice, we requested a single response from the most senior surgeon at the centre. It is likely, however, that possible differences between surgeons at each unit regarding operating time and opinions surrounding the importance of surgical factors were not included. Additionally, there is potential for selection bias due to the response rate (50%), although this is within typical email survey limits [29]. We believe the number and geographical spread of responses received sufficiently reflect the current UK practice.

This study provides a national overview of the current practice of UK plastic surgeons performing perineal reconstruction following APE/ELAPE. The information garnered can provide information regarding the scope of practice across the UK and the choice of reconstructive approach. It also highlights and ranks the importance of surgical factors involved in the reconstructive decision-making process. This is the first UK-based survey of perineal reconstruction following APE/ELAPE.

## Conclusions

Our national practice questionnaire demonstrates nationally well-established local networks between colorectal and plastic surgeons to reconstruct challenging perineal defects following APE/ELAPE. However, the variation in approaches reflects a lack of national consensus on the optimal reconstructive method, despite a vast majority of units employing a standardised reconstructive approach. We propose a wider collaboration to develop nationally agreed and evidence-based guidelines on the best practice in this field.

## Appendices

Appendix 1: Sample questionnaire sent out to units performing perineal reconstruction

General demographics

1.         Name of unit

a.         Free text.

2.         Geographical region

a.         South-West

b.         South-East

c.         North-West

d.         North-East

e.         Greater London

f.          Yorkshire and The Humber

g.         West Midlands

h.         East Midlands

i.          East of England

j.          Scotland

k.         Northern Ireland

l.          Wales

3.         Specialty of respondent

a.         Plastic surgery

b.         Colorectal surgery

4.         Grade of respondent

a.         Consultant

b.         Fellow

c.         SpR

d.         SHO

5.         Is there a plastic surgery unit available at your hospital?

a.         Yes

b.         No

Scope of practice

6.         How many perineal reconstructions following standard APE/ELAPE are performed at your unit every year?

a.         <10/year

b.         10-20/year

c.         21-30/year

d.         31-40/year

e.         41-50/year

f.          >50/year

g.         Other (enter free text)

7.         Do you have a dedicated APE/ELAPE service at your trust?

a.         Yes

b.         No

8.         Is there a standardised approach to reconstructing perineal defects following APE/ELAPE?

a.         Yes

b.         No

Choice of perineal reconstruction

9.         What is your preferred approach to reconstruction?

a.         Inferior gluteal artery perforator (IGAP) V-Y advancement flap

b.         Vertical rectus abdominus musculocutaneous (VRAM) flap

c.         Gluteal fold flap

d.         Profunda artery perforator (PAP) flap

e.         Transverse upper gracilis (TUG) flap

f.          Perineal turnover (PTO) flap

g.         Direct layered closure

h.         Interposition with biological mesh

i.          Other (Enter free text)

10.       In which position do you normally perform your reconstruction?

a.         Supine (Lloyd-Davies/lithotomy)

b.         Prone

c.         Other (enter free text)

11.       Do you routinely turn your patients intraoperatively?

a.         Yes

b.         No

12.       On average, what is the estimated operating time for the reconstruction of choice?

a.         <30 minutes

b.         30-59 minutes

c.         60-90 minutes

d.         91-120 minutes

e.         >120 minutes

f.          Other (enter free text)

13.       Do you routinely use mesh/acellular dermal matrix (ADM) for pelvic floor reconstruction?

a.         Yes

b.         No

Factors involved in decision-making

Please rank the importance of the factors below in the choice of reconstruction:

Size of defect

Previous radiotherapy

Patient habitus

Intraoperative positioning

Previous abdominal surgery

Cosmetic outcome

Other (enter free text)

Rank 1:

Rank 2:

Rank 3:

Rank 4:

Rank 5:

Rank 6:

Rank 7:

## References

[1] West NP, Anderin C, Smith KJ, Holm T, Quirke P (2010). Multicentre experience with extralevator abdominoperineal excision for low rectal cancer. Br J Surg.

[2] Holm T (2014). Controversies in abdominoperineal excision. Surg Oncol Clin N Am.

[3] Holm T, Ljung A, Häggmark T, Jurell G, Lagergren J (2007). Extended abdominoperineal resection with gluteus maximus flap reconstruction of the pelvic floor for rectal cancer. Br J Surg.

[4] Negoi I, Hostiuc S, Paun S, Negoi RI, Beuran M (2016). Extralevator vs conventional abdominoperineal resection for rectal cancer-a systematic review and meta-analysis. Am J Surg.

[5] Han JG, Wang ZJ, Wei GH, Gao ZG, Yang Y, Zhao BC (2012). Randomized clinical trial of conventional versus cylindrical abdominoperineal resection for locally advanced lower rectal cancer. Am J Surg.

[6] Lehtonen T, Räsänen M, Carpelan-Holmström M, Lepistö A (2019). Oncological outcomes before and after the extralevator abdominoperineal excision era in rectal cancer patients treated with abdominoperineal excision in a single centre, high volume unit. Colorectal Dis.

[7] Hainsworth A, Al Akash M, Roblin P, Mohanna P, Ross D, George ML (2012). Perineal reconstruction after abdominoperineal excision using inferior gluteal artery perforator flaps. Br J Surg.

[8] Peacock O, Pandya H, Sharp T, Hurst NG, Speake WJ, Tierney GM, Lund JN (2012). Biological mesh reconstruction of perineal wounds following enhanced abdominoperineal excision of rectum (APER). Int J Colorectal Dis.

[9] Bullard KM, Trudel JL, Baxter NN, Rothenberger DA (2005). Primary perineal wound closure after preoperative radiotherapy and abdominoperineal resection has a high incidence of wound failure. Dis Colon Rectum.

[10] Pollard CW, Nivatvongs S, Rojanasakul A, Ilstrup DM (1994). Carcinoma of the rectum. Profiles of intraoperative and early postoperative complications. Dis Colon Rectum.

[11] Rothenberger DA, Wong WD (1992). Abdominoperineal resection for adenocarcinoma of the low rectum. World J Surg.

[12] Butler CE, Gündeslioglu AO, Rodriguez-Bigas MA (2008). Outcomes of immediate vertical rectus abdominis myocutaneous flap reconstruction for irradiated abdominoperineal resection defects. J Am Coll Surg.

[13] Sinna R, Qassemyar Q, Benhaim T, Lauzanne P, Sabbagh C, Regimbeau JM, Mauvais F (2010). Perforator flaps: a new option in perineal reconstruction. J Plast Reconstr Aesthet Surg.

[14] Jones H, Moran B, Crane S, Hompes R, Cunningham C (2017). The LOREC APE registry: operative technique, oncological outcome and perineal wound healing after abdominoperineal excision. Colorectal Dis.

[15] Butt HZ, Salem MK, Vijaynagar B, Chaudhri S, Singh B (2013). Perineal reconstruction after extra-levator abdominoperineal excision (eLAPE): a systematic review. Int J Colorectal Dis.

[16] Prytz M, Angenete E, Ekelund J, Haglind E (2014). Extralevator abdominoperineal excision (ELAPE) for rectal cancer--short-term results from the Swedish Colorectal Cancer Registry. Selective use of ELAPE warranted. Int J Colorectal Dis.

[17] Dabbas N, Adams K, Chave H, Branagan G (2012). Current practice in abdominoperineal resection: an email survey of the membership of the Association of Coloproctology. Ann R Coll Surg Engl.

[18] Devulapalli C, Jia Wei AT, DiBiagio JR (2016). Primary versus flap closure of perineal defects following oncologic resection: a systematic review and meta-analysis. Plast Reconstr Surg.

[19] Nisar PJ, Scott HJ (2009). Myocutaneous flap reconstruction of the pelvis after abdominoperineal excision. Colorectal Dis.

[20] Sayers AE, Patel RK, Hunter IA (2015). Perineal hernia formation following extralevator abdominoperineal excision. Colorectal Dis.

[21] Jensen KK, Rashid L, Pilsgaard B, Møller P, Wille-Jørgensen P (2014). Pelvic floor reconstruction with a biological mesh after extralevator abdominoperineal excision leads to few perineal hernias and acceptable wound complication rates with minor movement limitations: single-centre experience including clinical examination and interview. Colorectal Dis.

[22] Johal KS, Mishra A, Alkizwini E (2022). Immediate vaginal and perineal reconstruction after abdominoperineal excision using the inferior gluteal artery perforator flap (V-IGAP). J Plast Reconstr Aesthet Surg.

[23] Nelson RA, Butler CE (2009). Surgical outcomes of VRAM versus thigh flaps for immediate reconstruction of pelvic and perineal cancer resection defects. Plast Reconstr Surg.

[24] Copeland-Halperin LR, Stewart T, Chen Y, Funderburk CD, Freed GL (2020). Perineal reconstruction following abdominoperineal resection: comprehensive review of the literature. J Plast Reconstr Aesthet Surg.

[25] Lutz BS, Khawaja S, Ingianni G (1997). Donor site morbidity after rectus abdominis muscle flaps. Eur J Plastic Surg.

[26] Antoniou SA, Agresta F, Garcia Alamino JM (2018). European Hernia Society guidelines on prevention and treatment of parastomal hernias. Hernia.

[27] Chasapi M, Maher M, Mitchell P, Dalal M (2018). The perineal turnover perforator flap: a new and simple technique for perineal reconstruction after extralevator abdominoperineal excision. Ann Plast Surg.

[28] Moura FS, Chasapi M, Mitchell P, Dalal MD (2021). Perineal turn over perforator flap: a novel surgical technique for combined perineal and posterior vaginal wall reconstruction. World J Plast Surg.

[29] Cook C, Heath F, Thompson RL (2016). A meta-analysis of response rates in web- or internet-based surveys. Educ Psychol Measure.

